# Adverse childhood experiences and adult stress eating

**DOI:** 10.21203/rs.3.rs-7623634/v1

**Published:** 2025-10-09

**Authors:** Sydney Aquilina, Martha Shrubsole, Julia Butt, Maureen Sanderson, David Schlundt, Mekeila Cook, Meira Epplein

**Affiliations:** Yale School of Medicine; Vanderbilt University; German Cancer Research Center; Meharry Medical College; Vanderbilt University; Meharry Medical College; Duke University

**Keywords:** Adverse childhood experiences, childhood trauma, eating behavior, stress eating

## Abstract

Adverse childhood experiences (ACEs) are an indicator of childhood trauma and are associated with worse adult health outcomes. We examined the association of ACEs and adult stress eating in a largely low-income and racially diverse population. Among 32,209 Southern Community Cohort Study participants, we used multivariable-adjusted logistic regression to estimate the association of ACEs with frequent adult stress eating. Individuals with any ACE were almost 50% more likely to report frequent stress eating as an adult compared to those without ACEs (OR: 1.47; 95% CI: 1.38, 1.56), and there was a significant dose-response trend with increasing number of ACEs. These associations remained significant after further adjusting for depression and emotional well-being. With the finding that individuals with ACEs are more likely to stress eat during adulthood, and both are implicated in a diverse array of poor health outcomes, further investigation of this association could have translational and therapeutic potential.

## Introduction

Adverse childhood experiences (ACEs) are linked to a growing number of poor physical and mental health outcomes throughout the human lifespan.^1–3^ A direct causal association is thought to exist between ACEs and psychological distress.^4^ Lasting changes in stress physiology, culminating in chronic stress, is thought to explain this phenomenon. Stress, especially chronic stress, is known to have many harmful health impacts. These include altered eating behaviors such as stress eating, a form of emotional eating.^5^ There is growing evidence suggesting stress eating increases risk of weight gain as well as one’s preference for high-fat and high-sugar (i.e. energy-dense) foods,^6^ which are associated with increased risk of a breadth of negative health consequences, including metabolic disease, gastrointestinal disease, liver disease, cardiovascular disease, and some cancers.^7–12^ Yet, to our knowledge, there has been no study exploring the relationship between ACEs and stress eating to date.

Therefore, we sought to examine the relationship between childhood trauma and frequent stress eating in adulthood. Specifically, we investigated whether being exposed to an ACE, and being exposed to multiple ACEs, increases the odds of frequent adult stress eating, and if differences exist by race and sex, in a large, racially diverse, prospective cohort study. As a result, this study aims to deepen our understanding of how the effects of ACEs manifest in adulthood, and the risk factors that are involved in adult stress eating. Elucidating high risk groups for stress eating has translational potential that could improve a range of health outcomes.

## Methods

### Study Population

From 2002 to 2009, the Southern Community Cohort Study (SCCS) recruited approximately 85,000 individuals aged 40–79 years old, primarily from community health clinics that serve the un- and under-insured, in 12 southeastern states, to create a prospective cohort.^13^ SCCS participants provided written informed consent, and protocols were approved by the Institutional Review Boards of Vanderbilt University and Meharry Medical College. For the present study, we included participants who: (1) completed the stress eating questions from the baseline questionnaire at enrollment; and (2) completed the ACE survey from the second follow-up questionnaire administered in 2012–2015.^13,14^

Of the 84,508 total SCCS participants, those with missing data for eating habits from the baseline questionnaire were removed (n=2,181). Additionally, because numbers were small for racial groups other than Black or White (n=4,123), and we were interested to see if associations differed within race-specific populations, we did not include them in the present analysis. Of the remaining 78,204, 38,226 completed the second follow-up questionnaire, generally reflecting the larger SCCS population.^13^ Among those who completed the second follow-up, 84.3% (n=32,209) completed the ACE questionnaire in full, such that only 15.7% (n=6,017) were excluded because they were missing a response to at least one of the ten ACE questions. Thus, we performed a retrospective cohort analysis including a total of 32,209 SCCS participants (19,868 Black individuals; 12,341 White individuals). Analyses were conducted among all and by self-reported sex and race in the following groups: Black females (n=13,196), Black males (n=6,672), White females (n=7,880), White males (n=4,461).

### Measures

An abbreviated version of the original ACE questionnaire, comprising ten questions, was used in this study. Each question reflects one of the ten identified ACEs: emotional abuse, physical abuse, sexual abuse, emotional neglect, physical neglect, parents divorced, mother physically abused, lived with an alcohol or drug abuser, had a household member who was mentally ill or attempted suicide, and had a household member go to prison.^15,16^ In accordance with the original ACE questionnaire, studies employing the condensed ACE questionnaire have found equivalent validity and wide-reaching associations, including cancer risk behaviors and health care utilization within the SCCS population.^14,16–18^

The question, “How often do you eat as a way to cope with negative feelings like anger, unhappiness, stress, or depression?” was used to measure the outcome, stress eating. There were nine possible responses: Never, Rarely, 1/month, 2–3/month, 1/week, 2–3/week, 4–6/week, 1/day, 2+/day. Based on response frequencies, responses were categorized as infrequent stress eating (less than 1/week) or frequent stress eating (1/week or more).

Covariates considered from the baseline survey included those related to either the exposure, ACEs, or the outcome, stress eating, including: age, body mass index (BMI), education, household income, smoking status, alcohol intake, depression diagnosis ever, current prescription for any anti-depressant/anti-anxiety medication, adverse adult experiences (emotional abuse, physical abuse, threatened with a weapon), emotional support figures, feeling helpless in the past month, feeling overwhelmed in the past month, and strength/comfort from religious faith. The baseline survey also included the 10-item Center for Epidemiologic Studies Depression Scale (CES-D 10).^19^ The CES-D 10 is a condensed version of the original 20-item Center for Epidemiologic Studies Depression Scale. In a 1994 evaluation of the abbreviated CES-D questionnaire, Andresen et al. found almost perfect agreement between the 20-item and 10-item versions (k=.97), and the CES-D 10 has since been widely regarded as a valid and reliable measure of depression symptoms.^19,20^ Additionally, the CES-D 10 does not include items 15, 17, or 19 from the CES-D 20 which have been called into question.^21^ As with the 20-item version, CES-D 10 participants must indicate a categorical response from a four-point ordinal scale: rarely or none of the time, some of the time, much of the time, most or all of the time.^19,21^ CES-D questions are designed to pertain to components of depressive symptomology (depressed mood, feelings of guilt and worthlessness, loss of appetite, sleep disturbance, feelings of helplessness and hopelessness, psychomotor deficit), and are divided into factors of positive and negative affect for scoring purposes.^20,22^ In order to estimate depression symptom severity, CES-D 10 score cutoffs of 10, 15, and 20 or higher are used for mild, moderate, and severe levels respectively.^19,23^ The distribution of symptom severity in the study population is displayed in Table 1.

For secondary stratification analyses, we created a binary depression variable, as previously used in this population,^24^ defined as having one or more of the following three characteristics: 1) a CES-D score of 15 or higher; 2) self-report of having been diagnosed with depression by a doctor; or 3) self-report of use of any anti-depression/anxiety prescriptions at the time of the baseline questionnaire. This combined, binary categorization of depression was used because we wanted to capture as many individuals who have been afflicted with depression in adulthood as possible, including those without adequate healthcare access or utilization. To note, we considered the possibility of including those with a CES-D score of 10 (mild symptoms) or higher to have depression in the combined depression variable. However, in order to avoid overly broadening the criteria and to ensure meaningful associations, only individuals with a CESD-10 score of 15 or higher (moderate to severe symptoms) were ultimately considered to be depressed for the purposes of this study.^19,23^

In addition, we created binary variables for adult adverse experiences (AAEs) and locus of control in order to account for the roles of post-childhood trauma and variations in one’s sense of control in life, respectively, in the pathway from childhood trauma to adult stress eating.^25^ Any AAE was defined as reporting one or more of three AAEs (emotional abuse, physical abuse, or threatened with a weapon), and having an internal locus of control was defined as reporting never or rarely to both the helpless and overwhelmed questions: (1) In the past month, how often have you felt that you were unable to control the important things in your life?; (2) In the past month, how often have you felt difficulties were piling up so high that you could not overcome them?

For further stratification analyses of the ACE-stress eating association, we additionally performed separate analyses dichotomizing for obesity (BMI ≥ 30) and diet quality (HEI-10 score ≤ 59.1867, the median score within the population), respectively.

### Statistical Analysis

The associations of baseline sociodemographic, lifestyle, and mental/emotional well-being variables with the outcome of interest, frequent adult stress eating, were assessed among all and in race/sex groupings, utilizing Pearson Chi-square tests for categorical variables, and t-tests for continuous variables. The distribution of most of these variables by ACE category has been previously described in the SCCS population, with findings of significant associations between ACEs and the majority of the adult socio-demographic and lifestyle variables (including younger age, lower household income, divorced or never married, smoking, and drinking).^26^

For our main analyses, logistic regression was performed to determine the association of ACEs with frequent stress eating in adulthood. We calculated odds ratios (ORs) and 95% confidence intervals (CIs) for frequent stress eating with the exposure of ACEs in four ways: by any ACE; by number of ACEs (in categories of 0, 1, 2, 3, 4+); by ACE category (abuse, neglect, household dysfunction); and with each of the ten individual ACEs. Having no ACEs at all, no ACEs for that category, and not having the individual ACE in question were used as the reference values. Stress eating frequency was considered in the same binary groups of *less often than weekly* and *weekly or daily*. In order to account for the impact of demographics on the association, all ORs were adjusted for age and sex as potential confounders, as well as income as a precision variable.

As a secondary analysis, we sought to determine the association between ACEs and frequent stress eating above and beyond the association with mental and emotional well-being. Thus, we calculated ORs adjusted for the impact of depression, having any adult adverse experiences, and locus of control (all as binary variables as described above), in addition to age, sex, and income.

To determine if the association of ACEs and stress eating was modified by adult exposures, we also performed stratified analyses by: (1) depression as a combined, binary variable; (2) current BMI, examining the association among obese individuals (BMI ≥ 30) and among non-obese individuals (BMI < 30); and (3) current diet quality as measured by the HEI-10 (dichotomized by median HEI score).

Lastly, we examined the association between the composite outcome of any ACE and any adult adverse experience (AAE) with frequent stress eating, using logistic regression. Having neither any ACE nor any AAE was used as the reference group, compared to: any ACE and no AAE; no ACE and any AAE; and any ACE and any AAE. ORs adjusted for the combined depression variable were calculated as an additional analysis.

## Results

Individuals reporting frequent stress eating were more likely to be younger, have a higher BMI, and have a lower household income (except White females) (Table 1). Notably, education was not associated consistently with frequent stress eating across the race-sex groupings. The association with smoking varied by race, whereby current smoking was associated with greater likelihood of stress eating among Black females and males, and never smoking was associated with greater likelihood of stress eating among White females and males. Additionally, individuals in all race-sex groups reporting frequent stress eating were more likely to: have ever been diagnosed with depression; currently be experiencing symptoms of depression; have a current prescription for anti-depressant/anxiety medication; have experienced adult adverse experiences (AAEs); have few emotional support figures; feel helpless or overwhelmed in the past month (external locus of control); and to receive less comfort or strength from religious faith (except White males).

For the main analyses, SCCS participants having any ACE were more likely to report frequent stress eating compared to those without ACEs (OR: 1.47; 95% CI 1.38, 1.56) (Table 2). This finding was consistent across all race/sex groupings, with ORs ranging from 1.35 (95% CI 1.12, 1.62) for Black males to 1.65 (95% CI 1.36, 2.00) for White males. These associations remained after adjusting for mental/emotional well-being both among all (OR: 1.15; 95% CI 1.07, 1.23) and in each race/sex group with the exception of Black females, for whom the fully adjusted OR did not reach statistical significance (OR 1.09; 95% CI 0.98, 1.21). Furthermore, individuals with increasing numbers of ACEs were more likely to report frequent stress eating compared to individuals with no ACEs, with ORs rising from 1.16 (95% CI 1.07, 1.25) for 1 ACE to 1.93 (95% CI 1.78, 2.09) for 4 or more ACEs (p for trend <0.0001). These associations also remained after mental and emotional well-being adjustment, with the exception of those with only 1 ACE. Similarly, whether categorized as abuse, neglect, or household dysfunction, any ACE was associated with frequent stress eating in every race/sex grouping after adjustment for demographics. After additionally adjusting for mental and emotional well-being, the associations remained among White males and females for all three ACE categories, but lost significance among Black males. Among Black females, the associations remained in the categories of abuse and neglect, but not household dysfunction. The individual ACEs with the strongest associations with frequent adult stress eating were living with a household member who was depressed or mentally ill or attempted suicide (OR: 1.76; 95% CI 1.63, 1.90), emotional neglect (OR: 1.69; 95% CI 1.57, 1.81), and emotional abuse (OR: 1.68; 95% CI 1.57, 1.80).

As depression may impact eating behaviors and have a disproportionate impact on the population by ACE score, we also considered the ACE-stress eating association separately among individuals not identified as having depression (n=20,924) and those with depression (n=11,285). Stratifying by the combined depression variable, ACEs were found to be associated with frequent stress eating both among those with and without identified depression (Table 3). Among those identified with depression, those with any ACE were found to have 21% higher odds of frequent stress eating than those without any ACEs (OR: 1.21; 95% CI 1.11, 1.32), with a significant trend of increasing odds of frequent stress eating with increasing number of ACEs (OR for 4 or more ACEs: 1.36; 95% CI: 1.22, 1.51; p for trend<0.0001). The association was particularly strong, however, among those without any identified depression, with a 29% increase in odds of frequent stress eating for individuals with any ACE compared to individuals with no ACEs (OR: 1.29; 95% CI 1.18, 1.41), reaching to a 53% increase in odds for those with 4 or more ACEs (OR: 1.53; 95% CI: 1.34, 1.76).

Categorizing individuals by both childhood and adult adverse experiences (ACEs and AAEs, respectively) also revealed associations with the odds of frequent stress eating (Table 4). SCCS participants were almost evenly divided among the No ACE/No AAE group (35%), the Any ACE/No AAE group (29%), and the Any ACE/Any AAE group (28%). Only 7% of our participants reported having experienced an AAE but not an ACE (No ACE/Any AAE group). Compared to having no ACE and no AAE, having either a childhood or an adult adverse experience was associated with over 30% increase in the odds of frequent stress eating as an adult (any ACE but no AAE OR: 1.31; 95% CI: 1.21, 1.41; no ACE but any AAE OR: 1.42; 95% CI: 1.27, 1.60). Having both any ACE and any AAE yielded an OR of 1.88 (95% CI: 1.74, 2.03). All three ORs remained significant even after adjustment for mental and emotional well-being. Furthermore, the strong finding of the increased odds of adult stress eating among those having both any ACE and any AAE was consistent for all race/sex-specific analyses.

The associations did not change when stratified by obesity or the Healthy Eating Index 2010 (Supplemental Tables 5 and 6).

## Discussion

In a large, diverse population in the southeastern US, we found that individuals who were exposed to adverse childhood experiences had higher odds of frequent stress eating in adulthood. This association exists for both Black and White men and women, persisting even after adjusting for both demographic factors and adult mental/emotional well-being factors. Equally intriguing, the ACE-stress eating association is neither affected by obesity nor diet quality, despite ACE-obesity and stress eating-obesity associations in the literature.^27,28^ These findings reveal the highly pervasive nature of the ACE-stress eating association and are underpinned by an expanding body of literature detailing associations between ACEs and unhealthy behaviors across a myriad of distinct populations.^29–32^ Even further, our results by number of ACEs suggest a dose-response effect.

Notably, the individual ACEs with the strongest associations with adult stress eating were different from those individual ACEs found to have the strongest associations with adult diet quality in a study of the same cohort.^26^ Specifically, we found that emotional neglect, emotional abuse, and living with a family member with mental health struggles produced the strongest associations with adult stress eating, whereas factors within household dysfunction were the strongest predictors of poor adult diet quality. This indicates that there may be differences in the long-term effects of particular ACEs which increase odds of certain poor health outcomes compared to others.

ACEs are known to be associated with poor mental health and depression, which are in turn known to influence eating behavior.^28,33,34^ Yet, our results indicate that the ACE-stress eating association is at least just as strong among those without identified depression as those with identified depression. This suggests depression may not explain the ACE-stress eating pathway, as ACEs increase odds of stress eating in adulthood irrespective of depression. Still, the association could be stronger among those without identified depression. This could be attributed to individuals with depression being more susceptible to not only emotional eating, but also other disordered eating, including appetite loss and undereating.^28^ Alternatively, individuals with ACEs but not depression may receive more comfort from stress eating than individuals with both ACEs and depression. This is supported by a 2015 study by Finch and Tomiyama, which found comfort eating to buffer an association between adverse life events in the past 12 months and perceived stress in 19-year-old adult females without, but not with, an elevated level of depressive symptoms.^35^ Individuals with ACEs may cope with their stress via stress eating depending on depression as a result.

Furthermore, this study found a statistically significant difference between the association with frequent adult stress eating among individuals with both any ACE and any AAE (OR: 1.88; 95% CI: 1.74, 2.03) and the groups having only either any ACE (OR: 1.31; 95% CI: 1.21, 1.41) or any AAE (OR: 1.42; 95% CI: 1.27, 1.60). Given the heightened odds of stress eating for individuals with both any ACE and any AAE, coupled with the vulnerability of individuals having any ACE with respect to AAE acquisition, there may be an opportunity for stress eating mitigation through trauma prevention initiatives even among adults with ACEs.

Although we had access to the full SCCS dataset, we did not have data on other variables that would have been useful to consider. These include, but are not limited to: intergenerational trauma,^36^ childhood variables (e.g. household income, eating practices), stress-induced undereating, eating disorders,^37^ self-compassion,^38^ positive experiences, and current life stressors. To note, data were also not collected regarding the types of foods eaten in order to cope with stress. However, because individuals in this population with ACEs are known to have poorer diet quality than those without ACEs,^26^ the foods consumed during stress eating are likely also less healthy (e.g. higher fat, higher sugar, higher calorie)^5^ among those with ACEs compared to those without ACEs. As a result, stress eating may be more harmful to the health of individuals with ACEs compared to individuals without ACEs.

Other limitations in this study also exist. For example, the CES-D questionnaire has contextual and structural limitations, such as question vagueness and the equal weighting schema of its items.^21^ Additionally, the CES-D is a measure of depressive symptoms at one point in time and would therefore not capture individuals who have had depressive symptoms in the past and are currently in remission.^22^ The ACE score is similarly an imperfect measure of the adverse experiences acquired during childhood. Since ACEs are self-reported, individuals must remember and recognize the adverse experience(s) from their childhood, which can be especially difficult for individuals who have experienced trauma. Given the possibility for an adverse experience during childhood to not fit into one of the screening questions, ACE scores may also be lower than the actual number of childhood adversities experienced, such that this study underestimates the odds of the association with stress eating. These issues extend similarly to the AAE questions. Furthermore, the outcome of stress eating was also self-reported. There are likely variations in self-awareness of stress eating, and the proportion of the population in this study that stress eats frequently may be higher than indicated by our data as a result.

However, there were notable strengths as well. For one, the large sample size (n=32,209) of this study enabled precise estimations of the association. Furthermore, the ACE questionnaire utilized was succinct to minimize survey fatigue. Another strength of this study was the AAE variable. Few studies on ACEs consider adverse experiences in adulthood, despite their potential to impact individuals similarly to ACEs.^39–41^ Additionally, we included ORs with and without adjustment for mental/emotional well-being factors. While the demographics-only adjusted ORs likely provide the best approximation of the true odds, the fully adjusted ORs demonstrate the pervasiveness of the association, namely that ACEs are associated with stress eating independent of depression, adult adverse experiences, and locus of control.

Importantly, there is limited research on the potential impacts of frequent stress eating.^35^ Future studies should explore potential short- and long-term health consequences as well as whether there are any benefits to this mechanism of coping. These impacts may depend on a variety of factors, including variables not available in this study, such as the type and variety of food(s) consumed.

Furthermore, there are likely numerous causal mechanisms driving the ACE-stress eating association.^25,42–44^ Additional studies are needed to delineate the pathway from childhood trauma to stress eating in adulthood, and ultimately determine how to intercede in this pathway. Future research should explore whether trauma interventions such as cognitive behavioral therapy, emotional eating interventions such as acceptance and commitment therapy,^45^ and stress reducing practices such as mindfulness meditation^46^ could disrupt the ACE-stress eating pathway and potential downstream health consequences. Interventions such as these should be explored both in childhood^47^ and adulthood, since earlier intervention may have a greater impact but be more difficult to employ.

Future studies should also explore whether locus of control interventions can decrease adult stress eating frequencies.^48^ Baseline locus of control may also be predictive of stress eating intervention efficacy. Studies investigating the efficacy of stress eating interventions should consider stratifying by ACE exposure. Special attention should also be given to other factors found to increase odds of stress eating in the models developed for this study, including female sex, depression, adult trauma, external locus of control, and having no or few emotional support figures.

While the fraction of daily to weekly stress eating found in this study is substantial, at 18% of the study population, and the relationship found between ACEs and stress eating did not change by obesity nor diet quality, research and intervention focus on stress eating frequencies should be supplemented by attention to types of foods consumed in stress eating. Providing access to healthier snacks, encouraging the purchase and consumption of healthier foods, and educating individuals on healthy eating practices may help mitigate the impact of stress eating on health.

In conclusion, ACEs are associated with frequent adult stress eating in this racially diverse and largely low-income population of the southeastern US, even beyond the impact of depression and other mental-emotional well-being factors. Thus, the present study suggests that individuals with ACEs are more likely to stress eat during adulthood. Since ACEs and stress eating are each implicated in a diverse array of poor health outcomes, further investigation of this association has translational and therapeutic potential.

## Supplementary Material

Supplementary Files

This is a list of supplementary files associated with this preprint. Click to download.

Tables.docx

## Abbreviations

AAE: adverse adult experience
ACE: adverse childhood experience
BMI: body mass index
CES-D 10: 10-item Center for Epidemiologic Studies Depression Scale
cpd: cigarettes per day
CI: confidence interval
HEI-10: Healthy Eating Index-2010
OR: odds ratio
SCCS: Southern Community Cohort Study
